# Detection of meningoencephalitis caused by *Listeria monocytogenes* with ischemic stroke-like onset using metagenomics next-generation sequencing: A case report

**DOI:** 10.1097/MD.0000000000026802

**Published:** 2021-08-06

**Authors:** Xiaobo Zhang, Ruying Wang, Jie Luo, Danni Xia, Chaojun Zhou

**Affiliations:** a Department of Neurology, The First People's Hospital of Changde City, Changde, Hunan, China; b Department of Laboratory, The First People's Hospital of Changde City, Changde, Hunan, China.

**Keywords:** cerebrospinal fluid, intracranial hemorrhage, *Listeria monocytogenes*, next-generation sequencing

## Abstract

**Rationale:**

*Listeria monocytogenes* (*L. monocytogenes*) is a compatible intracellular bacterial pathogen that can invade different mammalian cells and reach the central nervous system (CNS), leading to meningoencephalitis and brain abscesses. In the diagnosis of *L. monocytogenes* meningoencephalitis (LMM), conventional tests are often reported as negative due to antibiotic therapy or low bacterial content in cerebrospinal fluid. To date, prompt diagnosis and accurate treatment remain a challenge for patients with *Listeria* infections.

**Patient concerns:**

Here, we report a case of a 64-year-old male diagnosed with LMM by using metagenomics next-generation sequencing (mNGS).

**Diagnosis:**

LMM was confirmed by mNGS analysis of cerebrospinal fluid.

**Interventions:**

The patient was treated with piperacillin and sensitive antibiotics.

**Outcomes:**

The patient could walk independently about 1 month after admission and was discharged from the hospital.

**Lessons:**

This case highlights the value of mNGS in the diagnosis of LMM and emphasizes the inadequate sensitivity of conventional diagnostic methods for *Listeria* infection.

## 1 Introduction

Listeriosis is a foodborne zoonosis caused by *Listeria monocytogenes* (LM) and is common in people with low immunity. *L. monocytogene*s may cause meningitis, meningoencephalitis, and very rare complications, such as hydrocephalus and intracranial hemorrhage, which can cause high mortality and morbidity worldwide.^[1,2]^

The spectrum of microbial diseases has changed due to the increasing incidence of malignant tumors, acquired immunodeficiency syndrome (AIDS), and the use of immunosuppressive agents; in particular, the incidence of central nervous system (CNS) infections due to *Listeria* is on the rise, and it has become one of the common pathogens of bacterial meningitis.^[3]^ Some traditional detection methods, including isolation, culture, and identification, of pathogens in peripheral blood or cerebrospinal fluid have high operation requirement, long detection time, and low positive detection rate. Metagenomics next-generation sequencing (mNGS) is an emerging detection technique that improves the accuracy of diagnosis. mNGS has higher positive rate than traditional culture methods and can identify novel or rare pathogens within 1 to 2 days.^[4]^

## 2 Case report

Here, we report a case of a 64-year-old male diagnosed with *L. monocytogenes* meningoencephalitis (LMM) by using mNGS.

**Figure 1:**
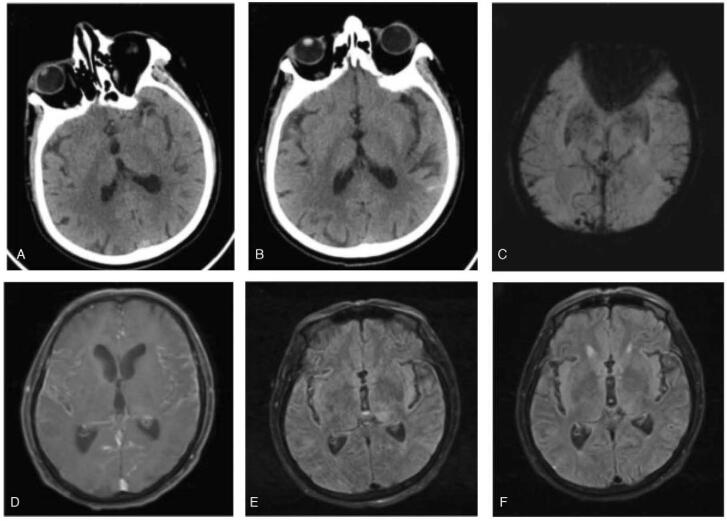
Imaging changes after the symptom onset (A) On the day of admission, CT indicates no bleeding. (B, C) On the 3th day following admission, CT and SWI indicates subarachnoid hemorrhage. (D, E) On the day of admission, MRI indicates extensive enhancement of the meanings, abnormal signals in the left thalamus and insula. (F) After 7 days of treatment, MRI indicates the thalamus and insula lesions were reduced. MRI = magnetic resonance imaging.

The patient was a farmer with a history of tuberculosis and transient ischemic attack (TIA). In October 2020, he was referred to our hospital due to sudden weakness of the right limb with facial twitching. He consumed long-term stored raw milk in the refrigerator 2 days before disease onset. On the day of admission, he was diagnosed with acute ischemic stroke due to bleeding on cranial CT (Fig. 1A). Further head CT-angiography confirmed no signs of cerebral artery stenosis or occlusion. Magnetic resonance imaging of the brain revealed extensive enhancement of the meanings as well as abnormal signals in the left thalamus and insula but not in accordance with infarction (Fig. 1D, E).

Subsequently, the patient developed a high fever in the afternoon of the day of admission. Physical examination showed positive meningeal irritation sign. Therefore, we completed the lumbar puncture examination immediately. The first lumbar puncture on admission revealed clear cerebrospinal fluid (CSF) with 150 leukocytes/mm^3^ (20% neutrophils, 80% monocytes; normal range, 0–10 leukocytes/mm^3^), 1680 mg/L protein (normal range, 150–450 mg /L), 1.8 mmol/L glucose (normal range, 2.5–4.5 mmol/L, plasma glucose 6.4 mmol/L), and pressure of 250 mmH_2_O. CSF stain was negative for fungi and Gram and acid-fast bacilli.

On the 3th day following admission, considering the aggravation of the patient's condition and persistent high fever and the unknown cause of infection, we completed the lumbar puncture examination repeatedly and sent cerebrospinal fluid and blood specimens to the laboratory for culture. At the same time, approximately 5 mL of CSF was sent to a third-party testing organization for CSF pathogen identification using mNGS. Repeated CSF examination showed bloody CSF with 330 leukocytes/mm^3^ (30% neutrophils, 70% monocytes), 18000 erythrocyte count/mm^3^, 2885 mg/L protein, 1.5 mmol/L glucose (plasma glucose 6.1 mmol/L), and pressure of 200 mmH_2_O. CSF stain showed Gram-positive rods and was negative for fungi and acid-fast bacilli. Subsequently, intracranial hemorrhage was also confirmed by CT (Fig. 1B) and susceptibility-weighted imaging (SWI) (Fig. 1C).

**Figure 2:**
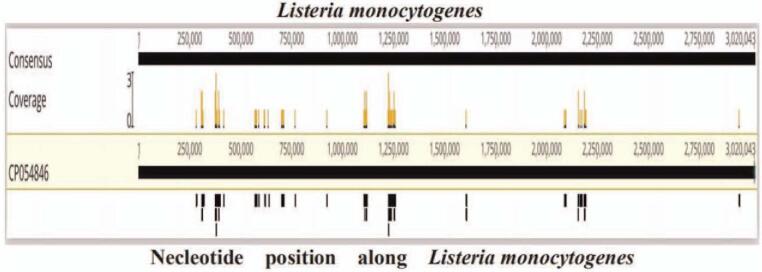
mNGS results of pathogen identification.

The patient was treated with piperacillin from the day of admission instead of anti-tuberculosis and anti-viral drugs. Facial convulsions did not recur, and limb motor function returned to normal. Magnetic resonance imaging re-examination confirmed that the thalamus and insula lesions were reduced (Fig. 1F). On the 7th day after admission, the mNGS result of CSF was suggestive of *Listeria* with a large number of sequences (Fig. 2). Two weeks after admission, the bacterial culture of CSF showed the presence of *L. monocytogene*s. Another lumbar puncture on the 16th day revealed turbid CSF with 34 leukocytes/mm^3^ (35% neutrophils, 65% monocytes), 330 erythrocyte count/mm^3^, 850 mg/L protein, 2.0 mmol/L glucose (plasma glucose 5.3 mmol/L), and pressure of 110 mmH_2_O, indicating a greater decrease in WBCs and protein. The results of CSF test conducted 3 times are shown in Table 1. During hospitalization, the blood laboratory findings showed normal tuberculosis and viral test results. Since then, we used antibiotics but not anti-tuberculosis and anti-viral drugs. The patient gradually improved in all aspects of physical condition. The patient could walk independently about 1 month after admission and was discharged from the hospital.

**Table 1:** Cerebrospinal fluid analysis across disease duration.

CSF test	On the first day	On the 3rd day	On the 17th day
Color	Clear	Bloody	Turbid
Pressure (cmH_2_0)	250	200	110
Erythrocyte count (/mm^3^)	0	18000	330
WBC count (/mm^3^) (normal range, 0–10)	150	330	34
WBC distribution (L/N)	80/20	70/30	65/35
Protein (mg/L) (normal range, 150–450)	1680	2885	850
CSF glucose (mmol/L) (normal range, 2.5–4.5)	1.8	1.5	2.0
Plasma glucose (mmol/L)	6.4	6.1	5.3
Gram stain	Normal	Gram-positive rods	Normal

### 2.1 Sample collection and data analysis

Approximately 3 to 5 mL of CSF was collected and sealed using a sterile technique. The sample was shipped on dry ice to IngeniGen XunMinKang Biotechnology Inc. (a third-party testing organization) in China for mNGS detection. This test uses high-throughput sequencing technology of Illumina, USA. We performed metagenomic analysis on the microbial DNA sequences in the samples, compared them with the microbial nucleic acid sequences in the database, and then identified the pathogenic microorganism. We used a double-ended 75 bp Illumina NextSeq platform for sequencing and detected a total of 9,597,156∗2 sequences (which can be understood as a data volume of approximately 19 M) for the sample. The database used was provided by Kraken. The range of detection covers the currently known pathogens including 7044 bacteria, 9233 virus, 2890 fungi, 172 parasites, 139 *Mycoplasma*, 128 *Chlamydia*, 102 *Rickettsia*, and 635 *Mycobacteria*. The lowest detection limit was 100 copies/mL, and the specificity was greater than 99.6%. A total of 118 reads were uniquely aligned to the *L. monocytogenes* genome.

## 3 Discussion

*L. monocytogene*s is an important foodborne pathogen that is mainly transmitted by the consumption of contaminated vegetables, animal products, and cheese; this pathogen invades the human body through the eyes and damaged skin and mucous membranes, mostly in perinatal women, newborns, and immunocompromised patients.^[5]^*L. monocytogene*s is neurotropic; once CNS infections occur, the mortality rate is high, especially when associated with hydrocephalus, brainstem encephalitis, and brain abscess, but bleeding in clinical practice is rare.^[6–8]^ The clinical presentation and CSF changes in LMM are similar to encephalitis by other causes, including viral meningitis and tuberculous meningitis.^[9]^ The consumption of contaminated foods is considered the main cause of listeriosis. In the present case, the consumption of raw milk stored in the refrigerator 2 days before the onset of illness provided the patient with conditions for infection with *Listeria*.

The patient was initially diagnosed with acute ischemic stroke but eventually diagnosed with intracranial infection with arachnoid hemorrhage after analysis of subsequent clinical symptoms and auxiliary examination. Considering that no pathogen was found by traditional detection methods, we performed mNGS analysis of CSF to determine *Listeria* meningitis for rapid and accurate diagnosis. To our knowledge, using mNGS of CSF for the diagnosis of intracranial hemorrhage due to *Listeria* meningoencephalitis has rarely been reported.

Initially, mNGS was used to diagnose CNS infections, such as chronic infections, and has been used to successfully diagnose rare encephalitis, new encephalitis, and atypical infections.^[10]^ For encephalitis cases of unknown cause or atypical symptoms, mNGS may be more advantageous than conventional methods, such as CSF smear and culture. In addition, mNGS is more suitable for implementation in the clinical practice for diagnosis of sepsis, severe infections in immunosuppressed hosts, severe pulmonary infections, infections with rare or new pathogens, and other infectious diseases.^[11]^ Many successful cases and studies have also demonstrated the strong potential of mNGS in the diagnosis of infectious diseases. Most articles have been published in the form of case reports, such as the use of mNGS to identify *Nocardia farcinica*,^[12]^*Streptococcus suis*,^[13]^ scrub typhus,^[14]^*Chlamydia psittaci*,^[15]^ and *L. monocytogenes*^[16]^ in specimens, while traditional methods have negative results.

## 4 Conclusion

This case highlights the value of mNGS in the diagnosis of LMM and emphasizes the inadequate sensitivity of conventional diagnostic methods for *Listeria* infection.

## Acknowledgments

The author is very grateful to the patient's family for agreeing to participate in this study.

## Author contributions

**Conceptualization:** Xiaobo Zhang.

**Writing – original draft:** Ruying Wang, Danni Xia, Jie Luo.

**Writing – review & editing:** Chaojun Zhou.
